# Dental Occlusion Measurement: A Methodological Evaluation to Improve Clinical Gnathology

**DOI:** 10.3390/s26165265

**Published:** 2026-08-20

**Authors:** Riccardo Rosati, Dolaji Henin, Gaia Pellegrini, Maria Francesca Sfondrini, Andrea Scribante, Daniela Carmagnola, Claudia Dellavia

**Affiliations:** 1 Department of Biomedical, Surgical and Dental Sciences, Università degli Studi di Milano, 20100 Milan, Italy; dolaji.henin@unimi.it (D.H.); gaia.pellegrini@unimi.it (G.P.); daniela.carmagnola@unimi.it (D.C.); claudia.dellavia@unimi.it (C.D.); 2 Unit of Orthodontics and Pediatric Dentistry, Section of Dentistry, Department of Clinical, Surgical, Diagnostic and Pediatric Sciences, University of Pavia, 27100 Pavia, Italy; francesca.sfondrini@unipv.it (M.F.S.); andrea.scribante@unipv.it (A.S.)

**Keywords:** standardized surface electromyography, dental occlusion, masticatory muscles

## Abstract

Dental-periodontal afferents contribute to modulating masticatory muscle recruitment. This modulation affects, in turn, load distribution within the stomatognathic apparatus. Standardized surface electromyography (ssEMG) has been proposed to quantify dental occlusion effects on muscle coordination. This study aimed to estimate the applicability of a research measurement protocol evaluating the repeatability and reproducibility of ssEMG-based indices acquired by non-expert clinicians. Sixty-five asymptomatic adults underwent ssEMG recordings of the masseter and anterior temporalis muscles, and 20 standardized indices were computed. Data were collected by non-expert operators during two sessions one week apart. Methodological reliability was assessed through intra- and inter-session ssEMG index comparisons, computing the coefficient of variation (CV%) and minimal detectable change (MDC). Differences in variability were also analyzed between subjects with typical and atypical functional indices. Overall, 15 out of 20 indices were considered clinically reliable. A statistically significant reduction in CV% was observed for five out of six clenching indices in subjects with typical values compared with those showing atypical patterns. ssEMG provides a reliable method for evaluating the functional effects of dental occlusion on masticatory muscle coordination by non-expert operators in routine clinical settings rather than research labs. These findings support the integration of functional, measurement-based approaches into evidence-based research and clinical gnathology.

## 1. Introduction

The masticatory system comprises a complex network of muscles that generate forces whose intensity, coordination, and direction determine the mechanical loads exerted on the jaw bones, temporomandibular joints, and dental arches, whether natural or prosthetic [1]. Repetitive teeth clenching or grinding (bruxism) has been associated with increased failure rates of implant-supported restorations [2], reduced clinical success of restorations in endodontically treated teeth [3], tooth displacement [4], and tooth wear [5].

Several studies have also suggested an association between repetitive jaw-muscle activity and temporomandibular disorders [6]. However, the most recent definition of bruxism [7] does not fully describe the muscle coordination patterns occurring during clenching episodes.

The recruitment of the masticatory muscles is influenced by several peripheral factors, including the distribution of occlusal contacts [8] and periodontal afferents, which are capable of detecting even minimal occlusal interferences [9]. Consequently, the interaction between occlusal morphology and muscle function is complex: patients with similar occlusal characteristics may exhibit different patterns of muscle recruitment, whereas individuals with different occlusions may display comparable neuromuscular behavior [10].

A better understanding of the influence of dental proprioception on masticatory muscle recruitment could improve both scientific knowledge and clinical management of functional overload. In particular, reliable assessment of the effect of dental occlusion on muscle coordination would help clinicians identify altered neuromuscular responses and optimize treatment strategies aimed at reducing excessive loading of oral structures.

Surface electromyography (sEMG) is a non-invasive and accessible technique that provides information on muscle function by recording the summed electrical activity of active motor units, thereby reflecting both muscle membrane properties and the control strategies of the central nervous system [11]. In dentistry, sEMG is currently used mainly in research settings to investigate the electrical activity of masticatory muscles, particularly the masseter (MM) and the anterior temporalis (TA) muscles [12,13,14,15].

Because sEMG does not directly assess individual muscle fibers, appropriate acquisition protocols should minimize both biological and technical sources of variability through standardized recording procedures and signal normalization (standardized surface electromyography, ssEMG) [16]. One of the most widely accepted normalization procedures consists of recording a maximum voluntary clench (MVC) on cotton rolls, which serves as the reference condition for subsequent recordings. The interposition of cotton rolls is assumed to minimize periodontal mechanoreceptor input. Consequently, differences between the sEMG activity recorded during the experimental tasks (e.g., clenching in intercuspal position, with oral appliances or dental prostheses, chewing, or swallowing) and that recorded during MVC on cotton rolls can be interpreted as reflecting the neuromuscular effect of dental occlusion under the tested condition.

ssEMG indices of the masticatory muscles are usually recorded during static clenching and dynamic chewing, on the right and left sides. The static evaluation assesses muscle activity during clenching in maximal intercuspation, and it may help to determine whether occlusal forces are evenly distributed across the dental arch or whether overload affects dental or joint structures, potentially leading to adaptive changes in muscle recruitment [11]. The dynamic evaluation, namely the chewing activity, allows quantification of muscle coordination patterns, as well as their frequency and variability, thus potentially allowing monitoring of the patient’s adaptive capacity to prosthetic or orthodontic rehabilitation during recovery.

The normalization procedure previously described is widely recognized as an effective method for improving the repeatability and reproducibility of masticatory sEMG recordings in both healthy individuals and dental patients [12,17,18,19,20], allowing reliable intra- and inter-subject comparisons of muscle recruitment patterns [11,21,22]. Nevertheless, the potential application of ssEMG in routine dental practice remains largely unexplored. To date, its use has been primarily confined to research settings, where examinations are performed by experienced operators under highly standardized conditions. However, ssEMG could represent a valuable clinical tool for objectively assessing patients’ functional status before, during, and after dental treatment. Demonstrating that the technique maintains adequate repeatability and reproducibility even when performed by non-expert operators would substantially enhance its translation into everyday clinical practice, where examinations are frequently conducted under less controlled conditions than those encountered in academic research. The aim of the present study was therefore to evaluate the repeatability and reproducibility of ssEMG recordings acquired under both static and dynamic conditions by non-expert operators, thereby assessing its potential for translation into routine clinical practice.

## 2. Materials and Methods

### 2.1. Study Design

In asymptomatic adults, ssEMG recordings of masticatory muscles were acquired twice by non-expert operators (undergraduate and postgraduate dental students) at a one-week interval (T1 and T2) during clenching and chewing tasks. Two acquisitions (A and B) were performed during each session, and data from T1 A, T1 B, T2 A and T2 B were compared to assess method repeatability and reproducibility.

### 2.2. Subjects

The study protocol was approved by the local Ethics Committee of the Università degli Studi di Milano (code: DG EMG 2016). Voluntary participants were recruited among undergraduate and postgraduate fellows attending the Dentistry degree of Università degli Studi di Milano and the Orthodontics specialty program of University of Pavia.

According to the inclusion criteria, all subjects were examined by an experienced dentist to ensure that they: (i) were in good general health; (ii) had no pathologies affecting the neck or the stomatognathic system; (iii) were pain-free during clenching; and (iv) presented a complete dentition up to the second maxillary and mandibular molars.

Exclusion criteria included the presence or history of temporomandibular disorders, according to the RDC/TMD examination protocol [23], periodontal unhealthy status [24], partial or total removable dental prostheses, and insufficient skin surface in the temporal area to allow the placement of bilateral electromyograph electrode pairs. All participants were informed about the experimental procedures and the potential risks associated with the instrumental examinations and provided written informed consent prior to participation.

### 2.3. Electrode Type and Positioning

The bilateral MM and TA muscles were examined. Disposable pre-gelled silver/silver chloride bipolar surface electrodes (H124SG, CardinalHealth™–Kendall™, Dublin, Ireland) were used for signal acquisition.

To ensure standardized and reproducible electrode placement, the anatomical references described by Ferrario et al. 2000 [11] were adopted. Electrode positioning was performed as follows:

- MM: With the subject seated, the operator stood in front of the participant and palpated the muscle belly during maximal tooth clenching. Electrodes were placed parallel to the exocanthion–gonion line, with the upper pole positioned below the tragus–labial commissure line.
- TA: The muscle belly was palpated during tooth clenching, and electrodes were positioned parallel to the exocanthion–gonion line along the anterior margin of the muscle, corresponding to the fronto-parietal suture.

To minimize skin impedance, the skin was carefully cleansed prior to electrode placement. Recordings were initiated five minutes after electrode application to allow adequate hydration of the skin by the conductive gel. ssEMG signals were acquired for further analysis, following a training session in which subjects learned to perform true maximal voluntary clenching using real-time ssEMG feedback.

Myoelectric activity of the MM and TA muscles was recorded using a computerized wireless electromyograph (Myowise, Cometa Systems, Milan, Italy), with four sensors directly connected to the surface electrodes and transmitting data via Bluetooth to the acquisition software (version 1.4, 1 February 2024, Cometa Systems, Milan, Italy). All five operators (under- and post-graduate dental students) received training from an experienced examiner on the proper execution of the instrumental assessment, consisting of a one-hour training session and an additional hour of supervised practical exercise, and the device user manual was provided for reference.

These sensors have a 2.4 GHz frequency with frequency-shift keying (FSK) modulation, a 1 Mbps data rate, −96 sBm sensitivity, a Flex PCB antenna (FlexiPCB, Cometa Systems, Milan, Italy) with an output power of 4 dB, filtered (high-pass filter set at 10 Hz, low-pass filter set at 500 Hz).

The analog ssEMG signal was amplified (gain 1000, bandwidth 0–1000 Hz, peak-to-peak input range from −3.3 to + 3.3 mV) using a differential amplifier with a high common mode rejection ratio (CMRR¼ > 120 dB typ.) and digitized (16-bit resolution, 1000 Hz A/D sampling frequency). The signals were averaged over 25 ms, with muscle activity assessed as the root mean square (RMS) of the amplitude (μV).

### 2.4. Normalization Procedure

Two cotton rolls (10 mm thick) were placed bilaterally on the mandibular second premolars/first molars, and a 5 s maximal voluntary contraction (MVC) was recorded. The mean ssEMG potential recorded during the first acquisition for each muscle was normalized to 100%, and all subsequent ssEMG potentials were expressed as a percentage of this value (µV/µV × 100) [11].

### 2.5. Analyzed Task

ssEMG activity was recorded during the following tasks:

- A 5 s maximal voluntary contraction (MVC) performed in dental intercuspation;
- 15 s of unilateral chewing on the right side;
- 15 s of unilateral chewing on the left side.

During the MVC test, subjects were instructed to clench as forcefully as possible and to maintain a constant level of contraction throughout the task. As in all previous studies adopting a similar methodology, the MVC test duration was set at 5 s to obtain enough EMG data for analysis through ssEMG indices while minimizing the risk of muscle fatigue [10,11,12,13,14]. Regarding the dynamic tests, the recording interval was extended to 15 s to allow the execution of a sufficient number of chewing cycles, enabling the calculation of dispersion statistics characterizing the population of masticatory movements [25,26,27]. During the chewing tasks, a softened sugar-free chewing gum was used.

### 2.6. ssEMG Data Analysis

Normalized ssEMG waveforms obtained during MVC and chewing tasks were analyzed by computing a total of 20 standardized ssEMG indices using the device proprietary software tools described below.

Clenching task indices (the 3s of the MVC recording with greater values were analyzed [11]).

(i) Activity Index (AC, %) [11], computed as the percentage ratio between the difference and the sum of the mean standardized ssEMG potentials of MM and TA, to identify the predominant masticatory muscle group. The index assumes positive values (up to +100%) when MM activity exceeds TA activity, negative values (down to −100%) when Temporalis activity predominates, and zero when both muscle groups contribute equally.
(ii) Asymmetry Index (AS, %) [11], calculated to evaluate the presence of unbalanced contractile activity between right and left muscles. The index ranges from +100% (complete prevalence of right-side muscle activity) to −100% (complete prevalence of left-side muscle activity).
(iii) Standardized Activity Index (Impact, %) [11], calculated to quantify the total muscular activity performed during maximal voluntary clenching relative to the standardized clenching on cotton rolls. Impact values were obtained by computing the mean total muscular activity (MM and TA), expressed as the integrated area of the ssEMG signal over time.
(iv) Percentage Overlapping Coefficient (POC, %) [11], an index of bilateral muscular symmetry. The POC ranges from 0% to 100%, with a value of 100% indicating perfect symmetry and synchronicity between paired muscles. POC values were calculated separately for MM and TA muscles for each subject.
(v) Torque Coefficient (TC, %) [11], used to assess unbalanced contractile activity between contralateral MM and TA muscles (e.g., right MM and left TA), which may generate a lateral displacing force component. TC values range from +100% (complete prevalence of right TA and left MM activity) to −100% (complete prevalence of left TA and right MM activity).

Chewing task indices (computed separately for right and left chewing):

(i) Ellipse (EL, %): the area of the confidence ellipse of the simultaneous differential left–right MM and TA activity (Lissajous plot) [25,26,27]. Each masticatory cycle is represented as a single point in a Cartesian coordinate system, with the x-axis corresponding to the differential left–right MM activity and the y-axis to the differential TA activity. From the set of coordinate pairs, Hotelling’s 95% confidence ellipse was calculated to assess the repeatability of the masticatory muscle contraction pattern. In subjects with normal neuromuscular coordination, under the assumption that the working side is more active than the balancing side, the centers of the ellipses describing unilateral chewing are expected to lie in the first (right-side chewing) and third (left-side chewing) quadrants of the Cartesian plane [25,26,27].
(ii) Acrophase (ACR, %): defined as the angle formed between the line connecting the center of the ellipse to the origin of the Cartesian plane. An ellipse located in the first quadrant (right-side chewing) exhibits an acrophase ranging from 0° to 90°, whereas chewing performed on the left side is expected to generate an ellipse located in the third quadrant, with an acrophase between 180° and 270° [25].
(iii) Modulus (MOD, %): is defined as the distance between the center of the Cartesian coordinate system and the center of the ellipse enclosing the coordinates of the masticatory cycles. An increasing difference between the standardized muscular activities of the two antimeric muscles results in a higher modulus [25].
(iv) Impact–Cycle (Impact/c, %): the global masticatory Impact coefficient (%), computed as in MVC/number of chewing cycles [27].
(v) Working-side Impact (WS Impact): computed as the percentage of the pooled muscle activity developed on the working side (right in the right-side chewing, left in the left-side chewing) [27].
(vi) Balancing-side Impact (BS Impact): computed as the percentage of the pooled muscle activity developed on the balancing side (left in the right-side chewing, right in the left-side chewing) [27].
(vii) Number masticatory cycles (N/cycles): resulting as the number of masticatory cycles performed in 15 s [27].

### 2.7. Statistical Evaluation

Statistical analyses were performed using R Statistical Software (version 4.5.2, 31 October 2025). The R packages used were RcppTOML, dotenv, stringr, readxl, ggplot2, tidyverse, moments, nortest, outliers, irr, patchwork, broom, kableExtra, bestNormalize, and qqplotr.

#### 2.7.1. Descriptive Statistics

The distributional properties of each index were evaluated using descriptive statistics, including the median and interquartile range (IQR), and coefficient of variation (CV%).

The AC, AS and TC indices associated with clenching activity are ranged between −100% and +100%, centered on zero. Consequently, their means approach zero, leading to an unstable and uninterpretable coefficient of variation (CV%), which uses the mean as the denominator. To address this limitation, the indices were shifted by 100 to eliminate negative values. This linear translation does not affect difference-based metrics, as the constant cancels out when computing differences between repeated measurements.

Prior to outlier removal, skewness and kurtosis parameters revealed that many variables had asymmetric distributions and significant correlations were found between age and the differences in two indices within the same session. Outlier removal was therefore performed to improve distributional properties. Instead of listwise deletion, outliers were removed on a case-by-case basis to preserve data integrity. Observations were flagged based on z-score, IQR, and the Grubbs’ test [28]. If an observation was identified as an outlier solely by the z-score or the IQR, it was retained (outliers list and values reported in Supplementary Tables S1 and S2 descriptive statistics for all standardized surface electromyography indices without outlier removal were reported).

Due to this outlier removal logic, sample sizes for reproducibility analyses were slightly larger than for repeatability analyses, as the latter required both repetitions within a session to be available, while the former used the mean of available repetitions per session.

Following outlier removal, distributional properties improved; however, non-normal indices were 48.8%, non-normal intra-session differences were 85%, while non-normal inter-session differences were 55% (Shapiro–Wilk test) [29]. Consequently, non-parametric tests and metrics were adopted for further analysis.

#### 2.7.2. Inferential Statistics

Subsequently, the repeatability and reproducibility of the indices were assessed. The differences between observations within each appointment, as well as the differences between the mean values obtained across different appointments, were calculated. The null hypothesis that the distribution of these differences is symmetric around zero was tested using the Wilcoxon signed-rank test with the Benjamini–Hochberg False Discovery Rate (FDR) correction [30].

Furthermore, the Minimal Detectable Change (MDC) was calculated.

Formulas used in statistical evaluation are reported in Table 1.

To assess whether the repeatability of ssEMG indices during clenching varies as a function of the indices themselves, a comparison of the CV% was performed between subjects with typical ssEMG indices, defined on the basis of the mean values reported in the available literature [19,27,31] and subjects with altered ssEMG indices. To compare the two groups for each index (typical vs. altered), the Mann–Whitney U test is adopted, as it does not assume a normal distribution for the intra-subject CV% metric and it is robust to outliers. The alternative hypothesis is that the distributions of intra-subject CV% differ between the two considered independent groups; the null hypothesis is that these are equal.

To assess whether the study sample size provided sufficient statistical power to demonstrate acceptable precision, a post hoc power analysis was conducted for both intra-session repeatability and inter-session reproducibility. The test evaluated whether measurement error was significantly below the 30% threshold (H0: CV% ≥ 30% vs. H1: CV% < 30%, alpha = 0.05). A nested cluster bootstrap (2000 resamples) was used to account for within-subject repeated measurements.

## 3. Results

A total of 65 subjects were enrolled: 26 males (mean age 26 years, SD 9 years) and 39 females (mean age 24 years, SD 3 years).

Descriptive statistics are reported in Table 2, and reliability metrics for all ssEMG indices (after outlier removal) are reported in Table 3. Table 4 reports CV% comparisons between subjects showing “typical” and “altered” ssEMG clenching indices.

All clenching indices showed mean values consistent with physiological muscle recruitment. AC, AS, and TC were centered around zero, indicating the absence of systematic lateralization or imbalance within the study population. POC values were high for both MM (84.5 ± 4.4%) and TA (85.6 ± 3.8%), indicating good bilateral muscular symmetry during maximal voluntary clenching. Impact averaged 101.2 ± 19.2%, confirming that clenching in maximal intercuspation generated muscular activation comparable to the standardized cotton-roll condition. During unilateral chewing, working-side muscular activity consistently exceeded balancing-side activity (approximately 61% vs. 39%) on both right and left chewing tasks, while the average number of chewing cycles was comparable between sides (22.8 ± 4.4 vs. 23.2 ± 4.2 cycles). Chewing-related indices, particularly Ellipse, Acrophase, and Modulus, showed greater inter-individual variability than clenching indices (Table 2).

The observed mean percentage differences between indices intra-session and inter-session never exceeded the MDC at the 95% confidence level, suggesting that the variability falls within the expected measurement error. Conversely, after establishing the CV% thresholds (excellent ≤ 10%, good ≤ 20%, acceptable ≤ 30%, poor > 30%), a subset of indices showed poor repeatability and reproducibility, with CV% exceeding the acceptable threshold. Clenching indices demonstrated the highest methodological reliability. Four of the six clenching indices (AS, POCMM, POCTA, and TC) achieved excellent repeatability and reproducibility, whereas AC and Impact reached excellent to good reliability. Among chewing indices, the poorest methodological performance was observed, in terms of repeatability, for right- and left-side Ellipse, while in terms of reproducibility, for Ellipse and Impact/Cycle on both chewing sides and for right-side Acrophase. All of these indices showed CV values exceeding the predefined acceptability threshold (Table 3).

Overall, 15 of 20 indices can be considered clinically and statistically reliable (CV% ≤ 30%). The remaining five indices should be further inspected.

Regarding the comparison of the CV% metric for each index between subjects with values within the typical range (Typical) and subjects with values outside the typical range (Altered), all analyzed indices were compatible with the typical range, except for the AC value, characterized by approximately 50% of values corresponding to those reported in the literature as reference values.

A statistically significant reduction in the percentage coefficient of variation was observed for five (AC, AS, POC MM, POC TA and TC) out of six indices in subjects with typical indices relative to those with atypical indices. Only the Impact value was non-significant (Table 4). However, due to the range of the POCMM and POCTA indexes (ranging between 0% and 100%), their corresponding CV% should be interpreted with caution. It is possible to observe, through the median and IQR of the two compared groups, that the medians of the “Typical” group are higher than those of the “Atypical” group for the AC, AS, POCMM, and POCTA indexes, while lower for the Impact and TC ones.

Results about statistical power are ranged between moderate to excellent (data summarized in Supplementary Figures S1 and S2), showing the maximum true CV detectable with at least 80% power alongside the observed CV for each index.

## 4. Discussion

The present study investigated the repeatability and reproducibility of ssEMG indices of the masticatory muscles acquired by non-expert operators during both static clenching and dynamic chewing. To the authors’ knowledge, this is the first study specifically designed to evaluate whether a standardized ssEMG protocol, originally developed for research purposes, can maintain adequate methodological reliability when transferred to a routine educational and clinical environment.

Overall, the results indicate that most ssEMG indices can be reliably acquired by undergraduate and postgraduate dental students, supporting the potential translation of this methodology from research laboratories to everyday clinical practice to effectively monitor healthy subjects’ neuromuscular adaptations to everyday dental treatments. The one-hour training session and an additional hour of supervised practical exercise performed in the present work aimed to prepare operators for the correct examination procedures, but no information about data interpretation was provided. If ssEMG-based occlusal evaluation is to be introduced into the dental curriculum, a more comprehensive training program would be required to ensure that participants gain an adequate understanding of both the methodology and the principles underlying data interpretation.

In a clinical application view, we also underline that the onset of temporomandibular disorders appears to be the cumulative consequence of multiple-system dysregulation [32] while baseline masticatory muscle asymmetry may predict treatment-related pain relief in temporomandibular disorders treatments [33]. Within this framework, the systematic and reliable monitoring of masticatory muscle function in asymptomatic individuals to identify muscular imbalances before symptom onset may represent a promising approach to improve clinical gnathology.

Concerning the static indices, the observed mean values and standard deviations were consistent with those previously reported in asymptomatic populations using the same standardized sEMG methodology [10,11,17,18,19,20,21,27,31,34,35,36,37,38,39]. Likewise, the dynamic ssEMG indices showed values comparable to those described in previous investigations. In particular, participants performed approximately 23 chewing cycles over 15 s (corresponding to a chewing frequency of approximately 1.5 Hz), closely matching the values reported by Ferrario et al. (2004), Dellavia et al. (2012), and Mapelli et al. (2016) [27,37,40]. The impact per cycle value (Impact–Cycle), which represents the energy developed during each chewing cycle as a percentage of the energy developed during clenching on cotton rolls, was also comparable with values reported in the literature. Previous studies reported mean values of 38% [12] and 45% [27,41], which are consistent with the mean value of 43.5% found in the present study (45% and 42% for the right and left sides, respectively). Similarly, the working side impact (WS Impact), approximately 60%, and the balancing side impact (BS Impact), approximately 40%, are in agreement with the results reported by the aforementioned researchers, supporting the validity of the proposed method. Regarding the dispersion of chewing coordination (Ellipse Index), values reported in the literature range from approximately 750 [27,40] to about 2000 percentage points [41], with the present study showing an intermediate mean value of around 1600. In all the cited studies, including the present one, this index shows high inter-subject variability, indicating that neuromuscular coordination during chewing differs considerably among individuals.

Overall, the agreement between the present findings and those consistently reported by several independent research groups supports the external validity of the standardized ssEMG methodology and indicates that recordings acquired by non-expert operators can reproduce the physiological characteristics of masticatory muscle function previously observed under controlled research conditions.

Based on the normal values reported in the literature, subjects were classified as presenting typical or altered functional indices according to whether their ssEMG values fell within or outside the published reference ranges. All clenching indices showed a clear predominance of subjects classified as typical, with the exception of the Activity Index that displayed an almost equal distribution of subjects classified as typical and altered according to the currently available reference values. This finding suggests that the reference interval proposed in previous studies may be relatively narrow, or alternatively that the physiological variability in the relative recruitment of the masseter and anterior temporalis muscles is greater than previously recognized [42]. Consequently, normal reference values for the Activity Index may require refinement through studies involving larger populations of healthy individuals.

The present findings demonstrated excellent methodological performance for the static clenching protocol. Four of the six clenching indices (AS, POCMM, POCTA and TC) showed excellent repeatability and reproducibility, whereas AC and Impact reached good reliability. From a clinical perspective, the results of this study indicate that an occlusal modification can be considered to alter muscle balance only if it produces changes greater than the MDC of the index being evaluated. For example, when testing an occlusal modification aimed at improving the asymmetry index, the change should be at least 10.9 percentage points to be considered real and not due to measurement variability. Conversely, the same conclusions cannot be drawn for the dynamic test, in which 2 out of 14 indices were not repeatable and 7 out of 14 were not reproducible when acquired by undergraduate and postgraduate dental students. This finding is not consistent with the repeatability data of dynamic analysis reported by Ferrario et al. 1991, 1996 [25,43]. A plausible explanation may be attributed to the greater complexity of dynamic mastication recordings, which require a higher level of operator expertise, particularly in adequately instructing participants to perform the test correctly and managing ssEMG signals artifacts due to skin movement during dynamic tasks. At first glance, these results might suggest that dynamic ssEMG analysis is less reliable than static clenching. However, the present findings indicate that this interpretation is probably oversimplified. Indeed, the lower repeatability observed for some chewing indices should not necessarily be interpreted as a limitation of the measurement technique itself. Rather, these indices appear to reflect the intrinsic biological variability of free mastication. Static clenching is a highly constrained motor task, requiring subjects to maintain maximal muscular contraction under standardized conditions. In contrast, chewing is a complex sensorimotor behavior continuously adapted by the central nervous system according to bolus position, periodontal sensory feedback and mandibular biomechanics. Consequently, physiological cycle-to-cycle variations cannot be completely eliminated, even when acquisition protocols are rigorously standardized. This interpretation is also supported by the different behavior of the dynamic indices. Parameters describing the global organization of chewing, such as the predominance of the working side over the balancing side, Working-side Impact, Balancing-side Impact and the number of chewing cycles, remained highly repeatable and reproducible. Conversely, indices describing the relative contribution of the masseter and temporalis muscles, such as Ellipse, Acrophase and, to a lesser extent, Modulus, exhibited considerably greater variability. The present results regarding muscle recruitment during dynamic chewing seem to confirm previous findings. For example, using three-dimensional kinematic analysis based on motion capture, Nilsson, Grip and Österlund [44] found a great variability in jaw movements, especially in the horizontal plane, emphasizing the natural biological movement variability. It overlaps with our results about muscle coordination during chewing, considering the temporalis and masseter biomechanical role in the mandible lateralization. Promising advances are emerging in muscular recruitment-articular movement relationships through the advanced evaluation of motor patterns using neuro-fuzzy inference system model-based sEMG analyses [45].

The study has several limitations. In particular, tests were conducted on asymptomatic subjects not requiring dental treatment, who were not recruited based on their dental condition. Indeed, the observed variability may be partly explained by the sample selection criteria used in this study, which focused on asymptomatic subjects without stratification based on morphological features that could affect the motor pattern, such as dental or skeletal occlusal class, occlusal plane divergence, or occlusal symmetry. Additionally, further studies on patients undergoing dental therapies are needed. Moreover, the effect of operator experience on the repeatability of ssEMG indices during dynamic mastication should be evaluated through control acquisitions performed by experienced operators. To facilitate the achievement of reliable ssEMG assessments, including during the dynamic mastication test, the Authors of the present study developed guidelines intended to support the application of functional dental occlusion analyses in clinical dental practice.

### 4.1. Practical Guidelines for ssEMG Data Acquisition

Based on the experience gained in the instrumental analysis of the masticatory muscles at the Functional Anatomy Research Center (FARC), University of Milan, the following operational objectives are proposed to facilitate operator training and improve the reliability of ssEMG recordings.

#### 4.1.1. Eligibility for Maximal Voluntary Clenching

Before data acquisition, it must be verified that the subject’s clinical condition allows maximal dental clenching without risk to oral structures. All hard and soft oral tissues should be healthy, free of infection, and structurally intact; subjects are free from pain at rest and able to clench comfortably, without pain, discomfort, or gag reflex.

#### 4.1.2. Acquisition Repeatability

Novice operators are recommended to initially focus on achieving repeatable acquisitions. Specifically, they should verify that:

(a) At least three calibration recordings are performed using cotton rolls, and the coherence of myoelectric signal amplitudes is evaluated. If coherence is inadequate, calibration should be repeated.
(b) As maximal clenching is a non-intuitive task, subjects should be allowed sufficient time to familiarize themselves with the motor task prior to instrumental recording.

#### 4.1.3. Remember the Functional Nature of the ssEMG Examination

The ssEMG analysis is a functional assessment aimed at measuring motor behavior rather than the morphology of static structures; moreover, functional assessment is not a one-shot examination but is derived from the evaluation of the mean of a (small or large) series of measurements.

(a) A minimum of two clenching recordings should be acquired and their coherence verified. In the absence of consistency, additional tasks should be acquired and the overall trend evaluated.
(b) When occlusal modifications are introduced (for example through provisional or definitive dental prostheses, orthodontic treatments, or gnathological interventions), and their effects on the masticatory muscles are to be evaluated, sufficient time should be allowed for a stable neuromuscular adaptation to the new occlusion to occur. In such cases, ssEMG recordings should be repeated several days after the occlusal modification in order to verify whether any immediate muscular adaptation remains stable over the following weeks or continues to evolve over time.

#### 4.1.4. Training on Well Balanced Subjects

(a) Initial recordings should be performed on subjects with normal muscular coordination patterns (i.e., all ssEMG indices within the reference range reported in Table 2) to develop technical consistency and repeatability.
(b) Reversible changes in dental occlusion can be tested by performing additional ssEMG recordings during clenching with paper strips placed between some teeth (or other reversible methods, i.e., a bite splint) to evaluate how subjects adapt, allowing classification of their functional response as low, medium, or high.
(c) In cases requiring occlusal modification for prosthetic or rehabilitative purposes, ssEMG should be initially applied to verify the preservation of comparable functional conditions before and after intervention in asymptomatic subjects

## 5. Conclusions

The present findings support the potential translation of ssEMG to routine clinical assessment of asymptomatic subjects undergoing dental treatments. The finding that reliable recordings can be obtained by non-expert operators suggests that objective assessments of the effects of occlusion on masticatory muscle coordination may become readily accessible in daily clinical practice, enabling more comprehensive patient evaluation, potentially supporting earlier identification of functional adaptations and dysfunctions.

## Figures and Tables

**Table 1:** Formulas used in statistical evaluation. i = Subject; a = Appointment (App1 or App2). MAD × 1.4826 is used as a robust estimator of σ, and the median is used for all across-subject aggregations to ensure robustness against heavy-tailed differences.

Formulas						
**Intra-session analysis**	**Diff %_a** = |median(diff_i,a)|/|median(mean_i,a)| × 100, where **diff_i,a** = rip1_i,a − rip2_i,a and **mean_i,a** = (rip1_i,a + rip2_i,a)/2			**Mean diff %** = (Diff%_App1 + Diff%_App2)/2		**Repeatable = (CV% ≤ 30%) AND (Diff% < MDC%)**
	**MDC95_a** = SEM_a × 1.96 × √2, where **SEM_a** = MAD(diff_i,a)/√2	**MDC %_a** = MDC95_a/|median(mean_i,a)| × 100			**Mean MDC %** = (MDC%_App1 + MDC%_App2)/2	
	**CV%_a** = (SEM_a/|median(mean_i,a)|) × 100		**Mean CV%** = (CV%_App1 + CV%_App2)/2			
**Inter-session analysis**	**Diff%** = |median(diff_inter_i)|/|median_inter| × 100, where **diff_inter_i** = mean_2,i − mean_1,i, **mean_a,i** = (rip1_i,a + rip2_i,a)/2, and **median_inter** = median [(mean_1,i + mean_2,i)/2:i = 1…n]					**Reproducible = (CV% ≤ 30%) AND (Diff% < MDC%)**
	**MDC95_inter** = SEM_inter × 1.96 × √2, where **SEM_inter** = MAD(diff_inter_i)/√2		**MDC%** = MDC95_inter/|median_inter| × 100			
	**CV%** = (SEM_inter/|median_inter|) × 100					

**Table 2:** Descriptive statistics for all standardized surface electromyography (ssEMG) indices (after outlier removal). Mean ± Standard Deviation (SD) and Median [Interquartile range (IQR)] were computed as the mean and median of subject-level averages across all available measurements. (Activity (AC); Asymmetry (AS); Standardized Activity Index (Impact); Percentage Overlapping Coefficient (POC); Torque Coefficient (TC); Ellipse (EL); Acrophase (ACR); Modulus (MOD); Impact–Cycle (Impact/c); Working-side Impact (WS Impact); Balancing-side Impact (BS Impact); Number of masticatory cycles (N/cycles)).

Masticatory Muscles ssEMG Indexes					
	Descriptive Statistics				
Index (%)	Subjects	Mean ± SD	Median [IQR]	Min	Max
**Clenching**					
Activity (AC)	63	−9.4 ± 15.3	−6.5 [−18.1, −0.3]	−56.7	22.4
Asymmetry (AS)	61	−1.7 ± 7.5	−1.7 [−7.1, 3.8]	−18.2	12.1
Standardized Activity Index (Impact)	61	101.2 ± 19.2	100.5 [89.1, 112.0]	36.8	150.5
MM Percentage Overlapping Coefficient (POC MM)	62	84.5 ± 4.4	85.6 [82.7, 87.7]	70.5	89.9
TA Percentage Overlapping Coefficient (POC TA)	60	85.6 ± 3.8	86.7 [84.2, 88.2]	72.5	91.3
Torque Coefficient (TC)	59	−0.6 ± 6.6	−1.1 [−4.3, 1.4]	−17.8	17.3
**Right chewing**					
Ellipse (EL)	61	1566.1 ± 1236.1	1245.0 [629.0, 2116.8]	130.4	6137.15
Acrophase (ACR)	64	96.0 ± 97.1	52.2 [28.7, 108.3]	7.7	354.4
Modulus (MOD)	62	106.4 ± 62.3	80.6 [61.2, 141.6]	10.1	92.6
Impact-Cycle (Impact/c)	61	44.7 ± 26.1	41.7 [25.0, 61.6]	6.0	132.8
Working-side Impact (WS Impact)	62	60.8 ± 6.9	59.4 [55.8, 63.9]	47.5	77.5
Balancing-side Impact (BS Impact)	62	39.2 ± 6.9	40.6 [36.1, 44.3]	22.5	52.5
Number masticatory cycles (N/cycles)	65	22.8 ± 4.4	22.5 [19.0, 25.8]	13.0	33.3
**Left chewing**					
Ellipse (EL)	63	1648.6 ± 1481.4	1272.8 [620.8, 2054.1]	62.3	6266.0
Acrophase (ACR)	62	218.0 ± 33.6	220.4 [202.8, 233.0]	101.1	307.1
Modulus (MOD)	63	117.0 ± 71.4	102.6 [64.4, 157.0]	18.5	370.6
Impact-Cycle (Impact/c)	60	42.3 ± 21.2	42.5 [26.9, 53.4]	7.5	86.4
Working-side Impact (WS Impact)	62	61.5 ± 7.8	61.3 [57.1, 65.8]	44.0	81.5
Balancing-side Impact (BS Impact)	64	38.5 ± 8.0	38.7 [33.9, 43.4]	18.5	56.0
Number masticatory cycles (N/cycles)	65	23.2 ± 4.2	23.5 [19.5, 25.8]	13.5	34.0

**Table 3:** Intra-session and inter-session repeatability metrics for all ssEMG indices (after outlier removal). For intra-session analysis, the pooled MAD was calculated across the two appointments by weighting the group MADs by their sample size; the pooled SEM (Standard Error of the Measurement) was derived as the ratio of the pooled MAD to the square root of 2, and the Minimal Detectable Change (MDC) and Coefficient of Variation (CV%) were computed from it, using the median as denominator. The absolute bias (Diff%) was calculated as the weighted median of intra-session paired differences across appointments, normalized to the pooled median. For inter-session analysis, MAD, SEM, Diff%, MDC, and CV% were calculated on the paired differences between the mean of the two repetitions per appointment. Confidence intervals (95%) for CV% and absolute MDC were established via non-parametric bootstrap (2000 resamplings), with the pooled MAD in the intra-session case computed by stacking the differences in both appointments within each bootstrap replicate. An index was classified as repeatable if the upper bound of the 95% CI for CV% was ≤30% (CV% thresholds: excellent ≤ 10%, good ≤ 20%, acceptable ≤ 30%, poor > 30%) and Diff% did not exceed the MDC% point estimate. In the repeatability case, the absence of a systematic order effect (*) was also evaluated using a Wilcoxon signed-rank test with Benjamini–Hochberg correction.

Masticatory Muscles ssEMG Indexes Reliability										
	Intra-Session Analysis					Inter-Session Analysis				
Index (%)	Diff %	Pooled MDC [CI 95%]	Pooled CV% [CI 95%]	CV % Quality	Repeatable	Diff %	MDC [CI 95%]	CV%	CV % Quality	Reproducible
**Clenching**										
Activity (AC)	0.2	10.25 [7.56–12.20]	3.9 [2.9–4.7]	Excellent	yes	3.7	32.9 [23.2–41.1]	7.5 [5.5–11.4]	Excellent/Good	yes
Asymmetry (AS)	0.0	8.21 [5.81–9.30]	3.0 [2.1–3.4]	Excellent	yes	0.9	18.5 [14.2–22.3]	4.2 [2.8–6.5]	Excellent	yes
Standardized Activity Index (Impact)	1.7	27.05 [20.34–36.90]	9.9 [7.4–13.4]	Good	yes	3.7	52.6 [37.7–66.5]	13.3 [7.9–18.1]	Good	yes
MM Percentage Overlapping Coefficient (POC MM)	0.2	4.22 [2.91–5.23]	1.8 [1.2–2.2]	Excellent	yes	0.1	10.8 [7.1–14.1]	1.9 [1.2–2.6]	Excellent	yes
TA Percentage Overlapping Coefficient (POC TA)	0.1	3.26 [2.62–4.36]	1.4 [1.1–1.8]	Excellent	yes	0.6	8.4 [5.9–10.5]	2.1 [1.3–2.8]	Excellent	yes
Torque Coefficient (TC)	0.1	5.58 [4.07–6.97]	2.0 [1.5–2.6]	Excellent	yes	0.1	16.3 [10.9–21.7]	5.0 [3.2–5.6]	Excellent	yes
**Right chewing**										
Ellipse (EL)	17.6	1123.33 [824.56–1549.51]	36.9 [26.5–49.4]	Poor	no	11.6	2754.0 [1818.3–3564.3]	37.6 [25.6–61.4]	Poor	no
Acrophase (ACR)	5.4	20.94 [16.56–30.80]	17.7 [13.4–28.6]	Good/Acceptable	yes	7.3	176.6 [109.7–228.5]	30.8 [17.8–40.0]	Poor	no
Modulus (MOD)	0.9	41.19 [30.80–50.56]	16.3 [11.3–21.3]	Good/Acceptable	yes	5	88.0 [64.9–107.3]	29.0 [18.2–37.1]	Acceptable/Poor	no
Impact-Cycle (Impact/c)	8.1	27.16 [18.02–33.56]	23.9 [15.7–28.7]	Acceptable	yes	2.2	66.7 [49.7–81.0]	30.7 [18.8–48.7]	Poor	no
Working-side Impact (WS Impact)	0.0	4.35 [2.91–5.81]	2.6 [1.7–3.6]	Excellent	yes	0.4	10.6 [7.9–13.1]	5.7 [3.9–7.2]	Excellent	yes
Balancing-side Impact (BS Impact)	0.0	4.35 [2.91–5.81]	3.9 [2.6–5.5]	Excellent/Good	yes	0.6	10.6 [7.9–13.1]	8.4 [5.8–10.7]	Excellent/Good	yes
Number masticatory cycles (N/cycles)	2.2	2.91 [2.91–2.91] *	4.6 [4.4–4.8]	Excellent	yes	4.4	5.0 [4.0–5.9]	7.0 [4.4–9.8]	Excellent	yes
**Left chewing**										
Ellipse (EL)	11.0	816.36 [633.18–1141.22]	25.1 [20.2–34.8]	Acceptable/Poor	no	13.5	3571.3 [1870.7–5320.1]	37.4 [28.6–55.5]	Poor	no
Acrophase (ACR)	0.6	16.22 [11.33–21.79]	2.7 [1.9–3.7]	Excellent	yes	1.1	58.8 [42.7–72.4]	4.4 [2.4–6.7]	Excellent	yes
Modulus (MOD)	0.9	28.54 [20.63–39.52]	10.0 [7.2–14.3]	Good	yes	3.1	95.7 [71.6–119.2]	22.5 [13.2–29.0]	Acceptable	yes
Impact-Cycle (Impact/c)	4.1	21.60 [13.95–29.35]	19.2 [12.1–25.6]	Good/Acceptable	yes	9.9	65.7 [50.9–78.3]	25.2 [14.9–46.4]	Acceptable/Poor	no
Working-side Impact (WS Impact)	0.0	2.91 [2.91–2.91] *	1.7 [1.7–1.8]	Excellent	yes	1.6	11.9 [9.1–14.5]	6.0 [3.4–8.3]	Excellent	yes
Balancing-side Impact (BS Impact)	0.0	2.91 [2.91–2.91] *	2.7 [2.6–2.8]	Excellent	yes	2.6	11.7 [9.1–14.1]	9.4 [5.5–12.3]	Excellent/Good	yes
Number masticatory cycles (N/cycles)	0.0	2.91 [2.91–2.91] *	4.5 [4.3–4.8]	Excellent	yes	4.3	6.1 [4.5–7.5]	8.9 [6.3–9.4]	Excellent	yes

**Table 4:** Comparison of the Coefficient of Variation (CV%) metric for each index between subjects with values within the typical range (Typical) and subjects with values outside the typical range (Altered). The CV% is estimated considering all four measures. The null hypothesis tested using the Mann–Whitney U test with Bonferroni correction is whether the CV% metrics are identical between the two groups. The CV% effect size is computed to evaluate the magnitude of the difference. The correlation coefficient, estimated with Spearman rank correlation, is also computed to assess the relationship between each subject’s distance from the typical range and their intra-subject CV%.

Masticatory Muscles ssEMG Variance: Typical and Altered Values Comparison												
Index (%)	Activity (AC)		Asymmetry (AS)		Standardized Activity Index (Impact)		MM Percentage Overlapping Coefficient (POC MM)		TA Percentage Overlapping Coefficient (POC TA)		Torque Coefficient (TC)	
	Typical	Altered	Typical	Altered	Typical	Altered	Typical	Altered	Typical	Altered	Typical	Altered
N	34	29	49	12	48	13	54	8	55	5	52	7
Median [IQR]	97.6 [91.5, 108.7]	81.8 [68.3, 95.3]	98.9 [90.7, 107.1]	88.4 [19.5, 97.3]	99.9 [85.8, 114.0]	127.0 [73.6, 180.4]	86.4 [82.6, 90.2]	74.7 [71.3, 78.1]	86.9 [83.6, 90.2]	76.8 [73.9, 79.7]	98.8 [93.6, 104.0]	110.7 [94.2, 127.2]
CV%	3.7	10.8	2.8	7.4	11.4	16.0	1.6	9.2	1.6	5.7	2.9	8.6
Effect size CV%	0.56		0.44		0.14		0.49		0.41		0.39	
*p*-value CV%	<0.001		0.004		>0.99		<0.001		0.01		0.017	
rho and *p*-value rho	0.61 (*p* < 0.001)		0.44 (*p* = 0.002)		0.15 (*p* = 1)		0.50 (*p* < 0.001)		0.41 (*p* = 0.006)		0.41 (*p* = 0.008)	
rho interpretation	Variance increases with the distance from the typical range		Variance increases with the distance from the typical range		No relationships		Variance increases with the distance from the typical range		Variance increases with the distance from the typical range		Variance increases with the distance from the typical range	

## Data Availability

The data that support the findings of this study are available from the corresponding author upon reasonable request.

## Supplementary Materials

The following supporting information can be downloaded at: https://www.mdpi.com/article/10.3390/s26165265/s1, Figure S1: Maximum true CV detectable with at least 80% power alongside the observed CV for each index for Repeatability; Figure S2: Maximum true CV detectable with at least 80% power alongside the observed CV for each index for Reproducibility; Table S1: List of removed outliers. Appointments (T1 and T2) and repetition (A and B) are specified; Table S2: Descriptive statistics for all standardized surface electromyography (ssEMG) indices (without outlier removal). Mean ± Standard Deviation (SD) and Median [Interquartile range (IQR)] were computed as the mean and median of subject-level averages across all available measurements.
